# A High Noise Immunity, 28 × 16-Channel Finger Touch Sensing IC Using OFDM and Frequency Translation Technique

**DOI:** 10.3390/s18051652

**Published:** 2018-05-21

**Authors:** SangYun Kim, Behnam Samadpoor Rikan, YoungGun Pu, Sang-Sun Yoo, Minjae Lee, Keum Cheol Hwang, Youngoo Yang, Kang-Yoon Lee

**Affiliations:** 1 College of Information and Communication Engineering, Sungkyunkwan University, Suwon 16419, Korea; ksy0501@skku.edu (S.Y.K.); behnam@skku.edu (B.S.R.); hara1015@naver.com (Y.G.P.); khwang@skku.edu (K.C.H.); yang09@skku.edu (Y.Y.); 2 Department of Smart Automobile, Pyeongtaek University, Pyeongtaek 17869, Korea; rapter@kaist.ac.kr; 3 School of Information and Communications, Gwangju Institute of Science and Technology, Gwangju 61005, Korea; minjae@gist.ac.kr

**Keywords:** analog front-end, capacitive touch panel, orthogonal frequency division multiplexing, low pass filter, variable gain amplifier, mixer

## Abstract

In this paper, a high noise immunity, 28 × 16-channel finger touch sensing IC for an orthogonal frequency division multiplexing (OFDM) touch sensing scheme is presented. In order to increase the signal-to-noise ratio (SNR), the OFDM sensing scheme is proposed. The transmitter (TX) transmits the orthogonal signal to each channels of the panel. The receiver (RX) detects the magnitude of the orthogonal frequency to be transmitted from the TX. Due to the orthogonal characteristics, it is robust to narrowband interference and noise. Therefore, the SNR can be improved. In order to reduce the noise effect of low frequencies, a mixer and high-pass filter are proposed as well. After the noise is filtered, the touch SNR attained is 60 dB, from 20 dB before the noise is filtered. The advantage of the proposed OFDM sensing scheme is its ability to detect channels of the panel simultaneously with the use of multiple carriers. To satisfy the linearity of the signal in the OFDM system, a high-linearity mixer and a rail-to-rail amplifier in the TX driver are designed. The proposed design is implemented in 90 nm CMOS process. The SNR is approximately 60 dB. The area is 13.6 mm^2^, and the power consumption is 62.4 mW.

## 1. Introduction

Recently, as the demand for the various touch applications has grown, the interest of touch technology has increased [1,2,3,4,5,6,7,8]. The touch system is composed of the touch panel and the touch sensing IC. In general, to detect the touch point, many types of touch panels, such as resistive, capacitive, and surface acoustic wave, are used to detect the touch point. Among many types of touch panels, the capacitive touch panel is adopted due to its advantages, such as the high optical quality, multi-touch capability, and so on [9]. In the area of the capacitive touch, the projected and mutual capacitance of the panel is used mostly. In this paper, the mutual capacitance touch panel is adopted to employ multi-touch and to reduce the ghost point [10].

In recent touch systems, time division has been used to process the touch signal [11,12,13]. In order to increase very small touch signals and reduce the noise, integrators are implemented in the receiver. By integrating the touch signal in capacitors, the touched small signal is amplified. In addition, to decrease the noise, the large capacitors of integrators are used because the integrators also have the role of a low-pass filter. This structure has a limit to increase the SNR in the analog front-end (AFE) and spends time to scan all of the channels in the touch panel. Additionally, the chip becomes large due to capacitors in the integrators. 

In this paper, to overcome the limit of the AFE, a frequency division sensing scheme is used. In general, when using multiple carriers, frequency division multiplexing (FDM) is widely used. To avoid the distortion of each frequency, the frequency band of the FDM is wide, whereas the frequency band of orthogonal frequency division multiplexing (OFDM) is small due to the orthogonal characteristic [14]. As the bandwidth of the OFDM is small, designing the AFE for the OFDM is easier than for the FDM. In addition, the various noises are rejected by orthogonality of frequencies and therefore the OFDM touch sensing scheme can acquire a high SNR in this system.

By using the OFDM sensing scheme, it is possible to detect each channel of the panel at the same time. Therefore, using the multiple-carrier sensing scheme, the scan rate can be increased. In addition, the proposed system can acquire time to average out noise by sensing one channel several times. 

**Figure 1:**
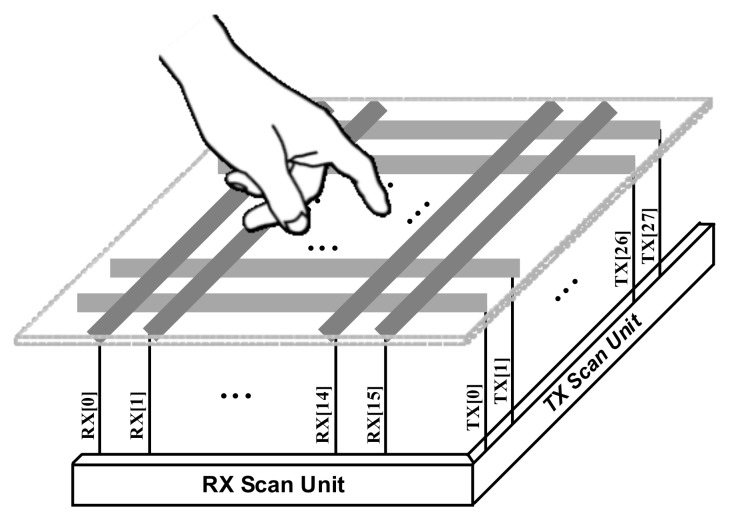
Finger touch system.

Figure 1 shows the conceptual diagram of the finger touch system. The transmitter (TX) of the AFE generates the OFDM pattern to the touch panel. The receiver (RX) of the AFE detects the received touch signal from the touch panel. When the touch is generated on the panel, the capacitor of the touch point is varied and the AFE senses the varied touch signal. After the signal is compared at each channel, the touch point is detected.

The suggested application is a capacitive touch panel and this panel is composed of 28 TX channels and 16 RX channels. This paper proposes the touch solution for the capacitive touch panel application. The rest of this paper is organized as follows: Section 2 describes the system description; Section 3 elaborates the design of the AFE architecture, including the building sub blocks; the AFE and its experimental results are discussed in Section 4, followed by conclusions in Section 5.

## 2. System Description

**Figure 2:**
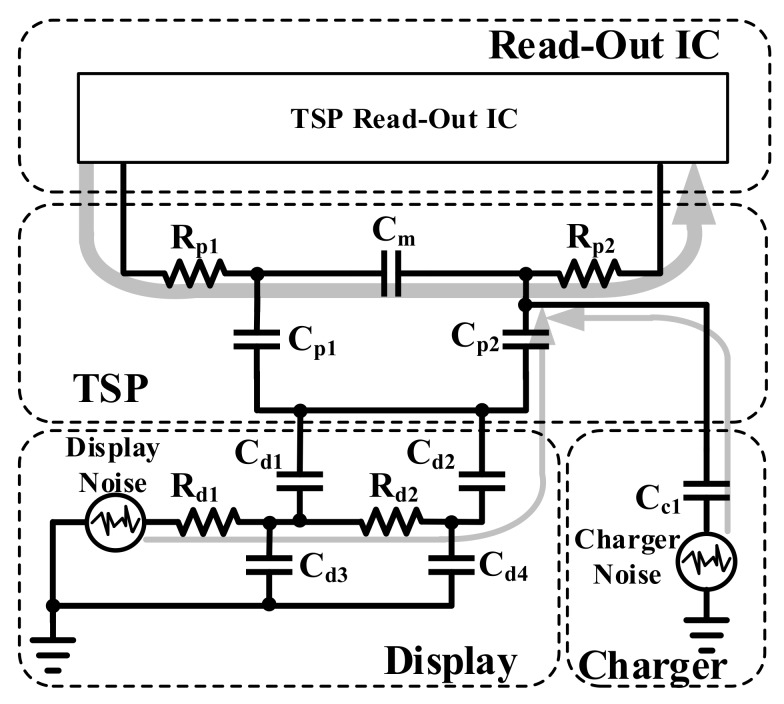
The circuit model and noise sources of the touch system.

Figure 2 shows the circuit model of the touch system. In the touch system, many kinds of noises are generated besides the touch screen panel (TSP). Among the variety of noises, the charger noise and display noise are dominant. Therefore, in order to improve the touch SNR in the read-out IC, it is important to reject them.

The charger noise is the noise that is generated from the battery charger. This noise is physically coupled into the TSP when there are touches. Due to this noise, performance factors, such as accuracy, can be degraded and the possibility of false or phantom touch can be increased. The typical characteristics of the charger, besides the TSP, are the frequency and amplitude of the charger noise, which are about 34.8 kHz and 2 Vpp, respectively. 

In electronic devices, the display noise can be conducted to the TSP directly because the TSP is fabricated on the display. Thus, the display noise can also degrade the touch performance. This noise is almost periodic from 10 kHz to 30 kHz and the amplitude is random from 500 mVpp to 3 Vpp [14]. In this paper, the touch sensing scheme and structure are proposed so that ambient noises, such as charger and display noises, are minimized.

**Figure 3:**
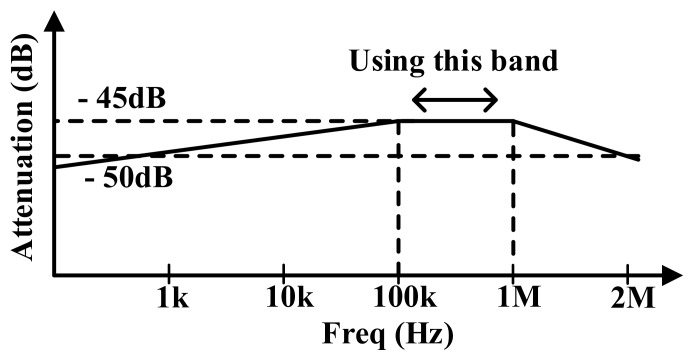
The characteristics of the panel model.

Figure 3 shows the characteristics of the panel model. The panel model has attenuations of −45 dB of 1 MHz and −50 dB at 2 MHz. The architecture of the AFE is determined by the characteristics of the panel in order to acquire an optimum signal processing spot. The attenuation of this panel has the characteristics of a bandpass filter. The flat band of the panel is from 100 kHz to 1 MHz. Therefore, to prevent the touch signal from decreasing in terms of the touch panel, the flat band of the panel is used.

**Figure 4:**
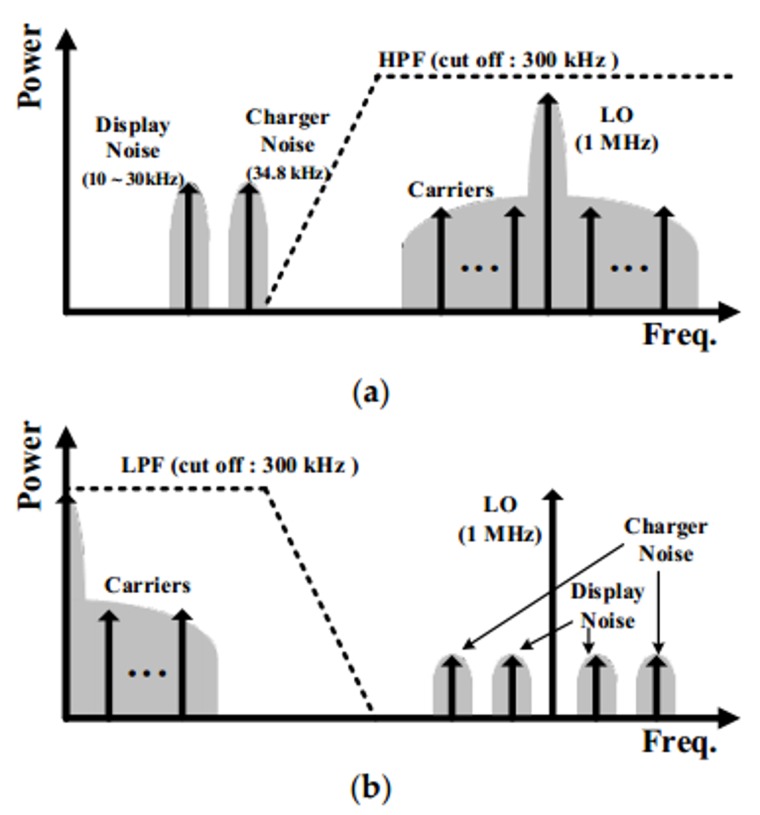
Spectrum of the touch signal and noises (**a**) before down-conversion and (**b**) after down-conversion.

Figure 4 shows the signal and ambient noise, such as the charger and display noises, in the frequency domain. Since the noises lie in the low frequency, the TX with the multiple carriers translates the baseband signals to high frequency to reduce the effect of the noise. Before the mixer down-converts the received signals to baseband signals, the high pass filter (HPF) in front of the RX can reduce the noise as shown in Figure 4a. After the noises are reduced by the HPF, the mixer of RX converts the received signal down to the baseband by eliminating the carriers, while the noises in the low frequency are converted into a high frequency. Therefore, the down-converted signal can be passed and the noises can be filtered by the LPF, as shown in Figure 4b. Through this mechanism, the noises are reduced and the touch signal can be acquired.

**Figure 5:**
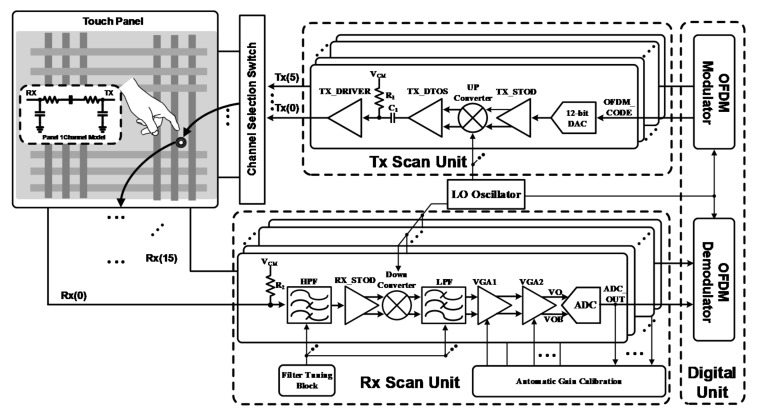
Block diagram of proposed analog front-end for the capacitive touch panel.

Figure 5 shows the block diagram of the capacitive touch panel AFE. The AFE is composed of the TX and RX scan unit. The TX scan unit consists of digital-to-analog converter (DAC), TX driver, and up-conversion mixer for driving the touch panel. The RX scan unit is composed of the HPF, single-to-differential converter (StoD), variable gain amplifier (VGA), low-pass filter (LPF) and analog-to-digital Converter (ADC). The TX scan unit generates the OFDM code through the DAC and this signal is converted to high frequency by the mixer. The mixed signal is transmitted to the capacitive touch panel by the TX driver. The mixer is used for the OFDM’s multi-carrier to avoid charger noise. The RX scan unit receives the signal to be converted to high frequency.

The RX scan unit receives the signal that is transmitted through the touch panel. In general, because the noise characteristic of the differential signal processing has a lower noise characteristic than that of single signal processing, the StoD is used to convert the single signal to a differential signal. To decrease the charge noise and low-frequency noise, the HPF is located in front of the RX scan unit. To pass the HPF, the bandwidth of the HPF is designed in consideration of the up-conversion frequency. After the signal from the HPF is changed from the single form to differential form, the mixer and the LPF converts the high to low frequency to recover the OFDM’s touch signal. The ADC converts the touch signal in order to modulate the OFDM. 

Contrary to the general AFE, the linearity and output dynamic range are important in the OFDM’s touch sensing scheme. Since the OFDM has multiple carriers, the linearity of the mixer and dynamic range of the amplifier are important. The mixer is designed as a high P1dB to obtain a high linearity. To reduce the current consumption and to prevent the distorted signal, the amplifier is designed as a rail to rail amplifier, which improves the dynamic range and a class AB amplifier is used. 

The AFE is composed of the 6th TX and the 16th scan unit to prevent bulky chip size and it can scan all of the touch panel channels by switching the MUX.

**Figure 6:**
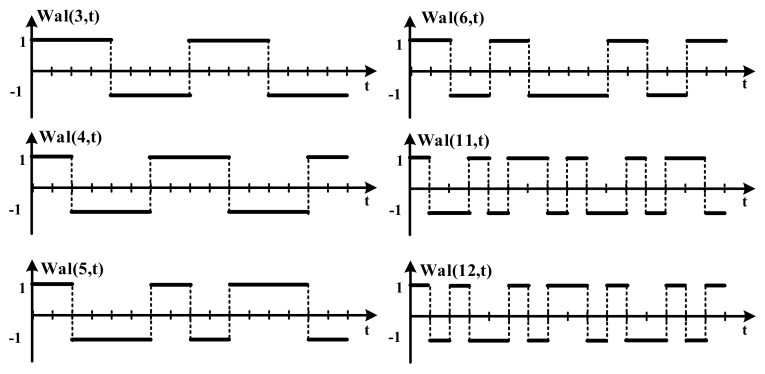
The used Walsh code in the OFDM system.

Figure 6 shows the Walsh code that is used. The formal Walsh functions are used in this system [15] and the channel is detected through the using the Walsh code with an orthogonal characteristic [16] in the OFDM system. In the Walsh function, as shown in Equation (1), when the Walsh code length is selected, the PAPR (peak to average power ratio) and orthogonality are considered. The shorter the code length, the more the PAPR is reduced [17]. The longer the code length, the more the orthogonality is improved. To acquire the orthogonality and low PAPR, the Walsh code length is optimized to 16. In the 16th channel’s code length, 16 kinds of the orthogonal signal can be obtained. In this system, only 6six kinds of orthogonal are selected because of the sixth channel’s TX design.

**Figure 7:**
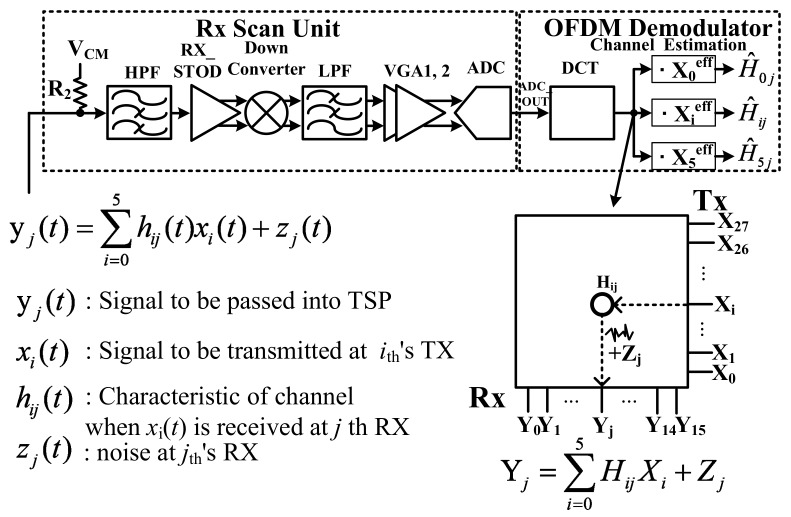
Conceptual diagram of the receiver.

Figure 7 shows the conceptual diagram of the RX. After the down conversion, the ADC converts the analog signal into digital. At each channel, the received signal from the TSP is reduced by the capacitance of the TSP and the noise is added from the TSP. The discrete cosine transform (DCT) block changes the time to the frequency domain to process the orthogonal frequency. The channel estimation can find the location of the channel from which the signal is generated. The channel estimation uses the orthogonality of the frequency. The *h_ij_*(*t*) and *z_j_*(*t*) represent the characteristics of the *i*th row and the *j*th column and the noise of the *j*th column from the TSP, respectively.

The *y_j_*(*t*) is the total signal to be received from the *j*th column channel of the TSP. The received signal is refined by the DCT block and channel estimation. In the OFDM demodulator, the requested frequency can be acquired by the orthogonality and the noise is rejected by the DCT and the channel estimation.

**Figure 8:**
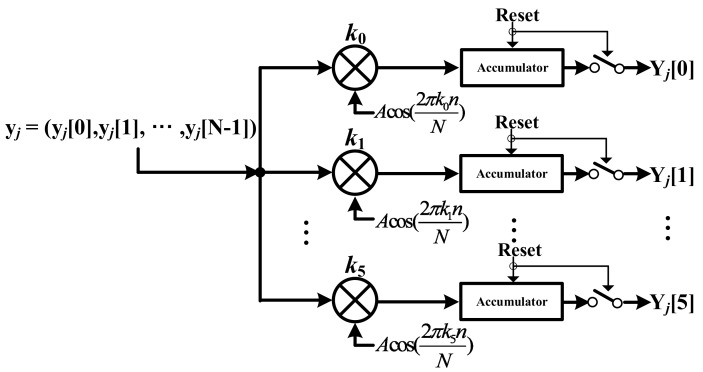
Block diagram of DCT.

Figure 8 shows the DCT block in the digital unit. In the digital unit, the point to be touched is detected by the OFDM scheme. In the AFE, the signal is transmitted to the digital unit using the orthogonal frequency. The digital unit loads the Walsh code at each frequency and the DCT block demodulates each code. The characteristics of the Walsh code means that if a different code is multiplied with the received code, the summation of each element in the Walsh code is zero. On the other hand, it is not zero if the same code is multiplied.

(1) $$\begin{array}{c} Wal\left(2^{m-1}+k-1,t\right)=x_{m}^{k}\left(t\right),\;\;\;\;\left[m=1,2,\ldots ,\;l=1,\ldots ,2^{m-1}\right]\\ x_{m+1}^{2k-1}\left(t\right)=\{\begin{array}{c} x_{m}^{k}\left(2t\right),\;\;\;\;\;\;\;\;\;\;\;\;\;\;\;\;\;\;\;\;\;\;\;\;\;\;\;0\leq t<0.5\\ {\left(-1\right)}^{k+1}x_{m}^{k}\left(2t-1\right),\;\;\;\;\;0.5\leq t<1 \end{array}\\ x_{m+1}^{k}\left(t\right)=\{\begin{array}{c} x_{m}^{k}\left(2t\right),\;\;\;\;\;\;\;\;\;\;\;\;\;\;\;\;\;\;\;\;\;\;\;\;\;\;\;0\leq t<0.5\\ {\left(-1\right)}^{k}x_{m}^{k}\left(2t-1\right),\;\;\;\;\;\;\;\;\;\;0.5\leq t<1 \end{array} \end{array}$$

As with the use of orthogonality, the noises of several frequencies in the panel are rejected and, therefore, the SNR is also improved. 

The OFDM scheme uses the DCT. The DCT block receives the signal *y_j_* = (*y_j_*[0], …, *y_j_*[N − 1]) from the ADC. *Y_j_*[0], *Y_j_*[1], …, *Y_j_*[5] are only acquired through the DCT block because this OFDM system only uses six orthogonal frequencies as the subcarrier. The DCT block uses Equation (2), as follows:

(2) $$Y_{j}\left[k\right]=\overset{N-1}{\underset{n=0}{\sum }}y_{j}\left[n\right]\cdot \cos\left(\frac{2\pi }{N}kn\right),\;\;\;k=0,1,\ldots ,N-1$$

(3) $$\begin{array}{l} {\hat{H}}_{ij}=Y_{j}^{eff}\cdot X_{i}^{eff}/{\left|\left|X_{i}^{eff}\right|\right|}^{2}\\ \;\;\;\;\;\;=\left(Y_{j}\left[0\right],Y_{j}\left[1\right],\ldots ,Y_{j}\left[4\right]\right)\cdot \left(X_{i}\left[0\right],X_{i}\left[1\right],\ldots ,X_{i}\left[4\right]\right)/{\left|\left|X_{i}^{eff}\right|\right|}^{2}\\ \;\;\;\;\;\;=\left(H_{1j}X_{1}^{eff}+\ldots +H_{6j}X_{6}^{eff}+Z_{j}^{eff}\right)\cdot \left(X_{i}\left[0\right],X_{i}\left[1\right],\ldots ,X_{i}\left[4\right]\right)/{\left|\left|X_{i}^{eff}\right|\right|}^{2}\\ \;\;\;\;\;\;=\left(H_{ij}{\left|\left|X_{i}^{eff}\right|\right|}^{2}+Z_{j}^{eff}\cdot X_{i}^{eff}\right)/{\left|\left|X_{i}^{eff}\right|\right|}^{2}=H_{ij}+Z_{j}^{eff}\cdot X_{i}^{eff}/{\left|\left|X_{i}^{eff}\right|\right|}^{2}\\ \;\;\;\;\;\;\;\;\;\left(∵\mathrm{if}\;i\neq j,\;X_{i}^{eff}\cdot X_{j}^{eff}=0\;by\;orthogonality\right) \end{array}$$

**Figure 9:**
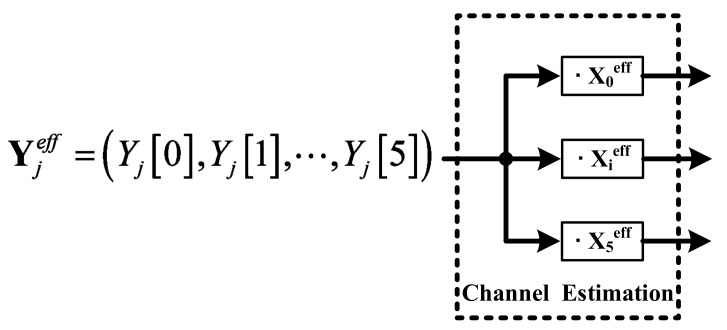
The concept of the channel estimation.

Figure 9 shows the concept of the channel estimation. The channel estimation can detect the channel by using the orthogonality of the frequency. Since each carrier comprises orthogonal characteristics, this system can find the channel by using the inner product calculation from Equation (3). Equation (2) presents the channel estimation equation. The index $X_{i}^{eff}$ = (X*_i_*[0], …, X*_i_*[5]) to relate with the orthogonal frequency is included in the channel estimation. With the inner product of this index, the Ĥ*_ij_* is generated, and this system can recognize the channel of the touch panel.

## 3. Proposed Analog Front-End Architecture for a Capacitive Touch Panel

### 3.1. Transmitter

**Figure 10:**
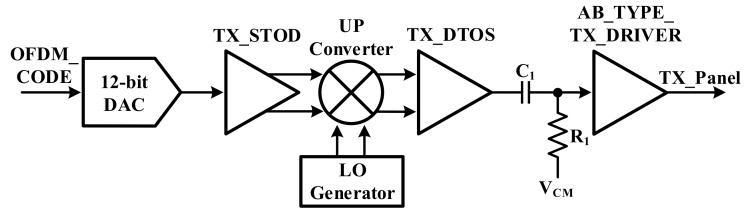
Architecture of transmitter.

Figure 10 shows the transmitter architecture of the touch panel. It is composed of the DAC, StoD, mixer, and TX driver. The DAC is used to convert the OFDM code to an analog signal. The converted OFDM analog signal is converted from a low to a high frequency by using the mixer. Due to the characteristic of the capacitance of the touch panel, the OFDM analog signal is transmitted to the touch panel by the TX driver. In the designed transmitter, in order to reduce the noise and to avoid the charger noise, differential signal processing in the designed transmitter is adopted and an up-conversion mixer is proposed.

### 3.2. Transmitter Mixer

**Figure 11:**
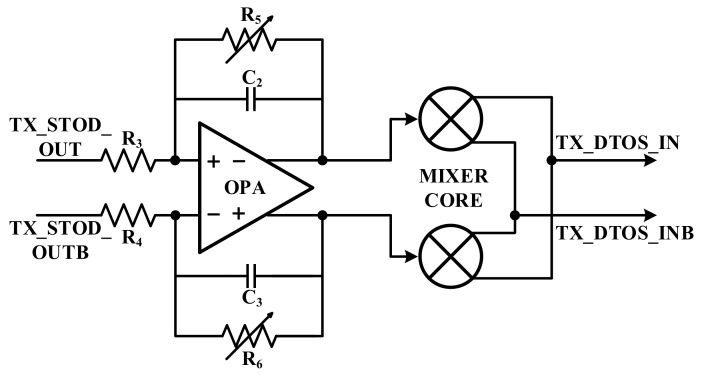
Block diagram of transmitter mixer.

Figure 11 presents the transmitter mixer. The characteristic of the OFDM sensing scheme must be considered in this transmitter mixer. In order to acquire a high SNR, a large signal has to be transmitted to the touch panel. For this reason, the AFE must have the ability to drive a high dynamic range. In addition, when the signal is transmitted to the touch panel, the linearity has to be guaranteed. The linearity and removal of the PAPR disadvantage are important in terms of the OFDM sensing scheme. If the linearity is broken in this system, it is difficult for the OFDM modulator to detect the touch point because the carriers are distorted by the AFE. Therefore, the P1dB of the mixer is important in this design.

To acquire linearity, the passive-type mixing stage has been selected. Since the gain of the passive mixer is low, the gain stage is designed in front of the passive mixer to increase the gain. 

**Figure 12:**
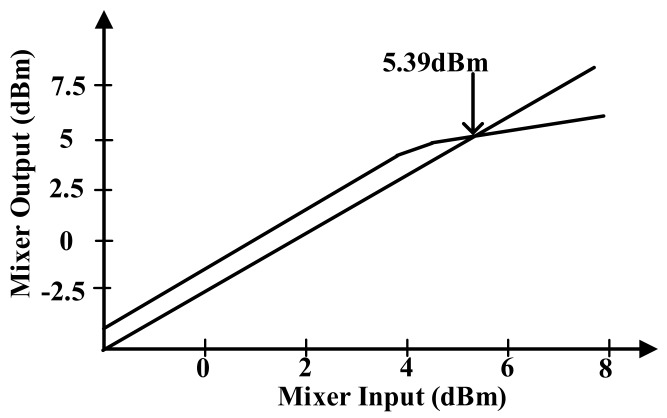
P1dB simulation of transmitter mixer.

Figure 12 presents the P1dB simulation result. The proposed mixer guarantees the linearity until 5.39 dBm because the DAC generates a 1.2 Vpp OFDM signal.

### 3.3. Transmitter Driver

**Figure 13:**
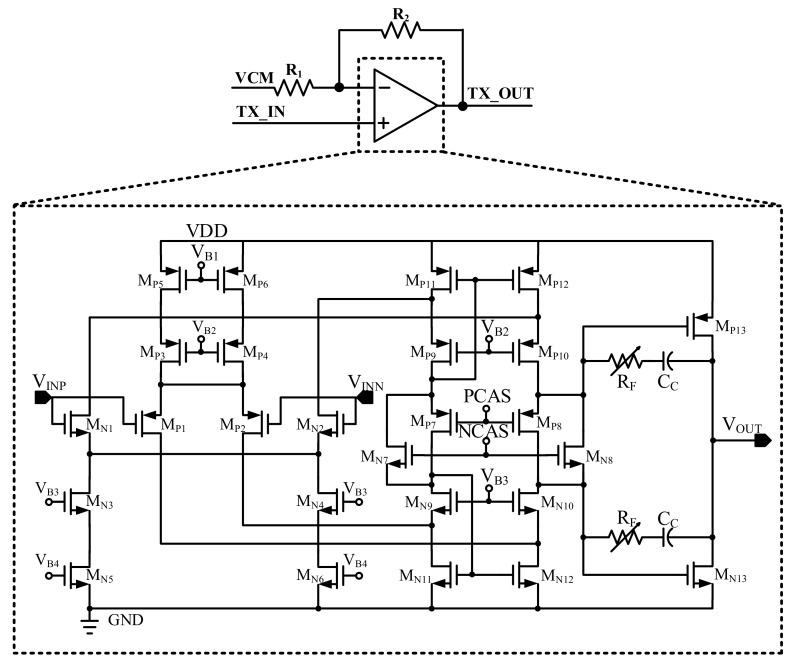
Transmitter driver.

Figure 13 shows the transmitter driver that drives the capacitive touch panel. It is composed of the AB type amplifier and resisters. This driver delivers the signal as following the ratio of R_1_ and R_2_. Especially, the proposed driver consists of higher voltage metal oxide silicon field effect transistor (MOSFET) than that of the other designed block. Since the TX signal is reduced when it passes through the touch panel, it is necessary to increase the TX signal. For the acquisition of a high SNR, only this block is designed as a high-voltage MOSFET.

The amplifier to be used in transmitter driver is class AB type amplifier. In this TX driver, the linearity in the OFDM sensing scheme, current consumption in the AFE and the panel driving ability is considered. In addition, in order to acquire the accuracy and to reduce the high gain, a PMOS input type amplifier is adopted. The characteristics of the class A type is that the signal distortion is lowest and the current consumption that is greater than those of the other classes of amplifiers. In the case of the class B type amplifier, the current consumption is low.

However, because of the push-pull type amplifier, crossover distortion is generated and the linearity is not good. In order to exploit both class A and B advantages, the class AB type amplifier is used in the transmitter driver. As this type amplifier operates at a conduction angle between 180° and 360°, the crossover distortion is minimized and the linear characteristic is improved. In addition, the current consumption is reduced blow that of the class A type [18]. Therefore, through the adoption of the class AB type amplifier, the linearity of the OFDM sensing scheme, the minimized current consumption of the AFE, and the touch panel driving ability are obtained.

### 3.4. Receiver

**Figure 14:**
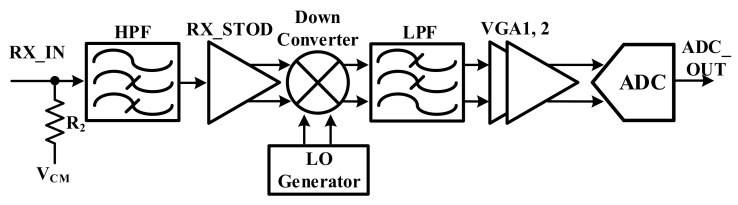
Architecture of the receiver.

Figure 14 shows the receiver architecture of the touch panel. It is composed of a HPF, StoD, mixer, LPF, and VGA. To increase the SNR and reduce the effect of the low-frequency noise, the HPF and the differential signal processing AFE are adopted. As the transmitter delivers the upconversion frequency to the touch panel, the receiver mixer converts the frequency from high to low frequency by the receiver mixer so that the OFDM sensing scheme can be used in the demodulator.

In general, because the signal that is passed from the touch panel is too small, the VGA is needed to increase the signal. The designed 2 dB and 1 dB steps of the VGA compensates the gain mismatch at each AFE channel. After the VGA increases the analog signal, the ADC processes the analog-to-digital conversion to deliver the signal to the OFDM demodulator.

### 3.5. Receiver Mixer

**Figure 15:**
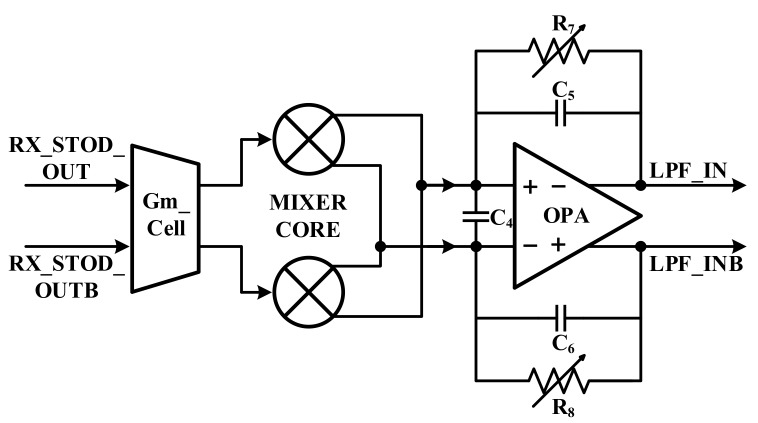
Block diagram of the receiver mixer.

Figure 15 shows the receiver mixer that is used in the receiver. To acquire the transmitter-mixer-derived linearity, the passive type mixer is used. Due to the mixer input, the TX and RX mixer structures are different. The transmitter mixer that is close to 0 dB VGA is attached in front of the mixer. Since the input of the transmitter mixer is too large, the amplification of the signal is not needed and the P1dB is too high. If the TX mixer is used in the receiver, the signal of the receiver cannot be amplified. Therefore, the receiver mixer needs that the Gm cell can increase the signal and drive the current.

**Figure 16:**
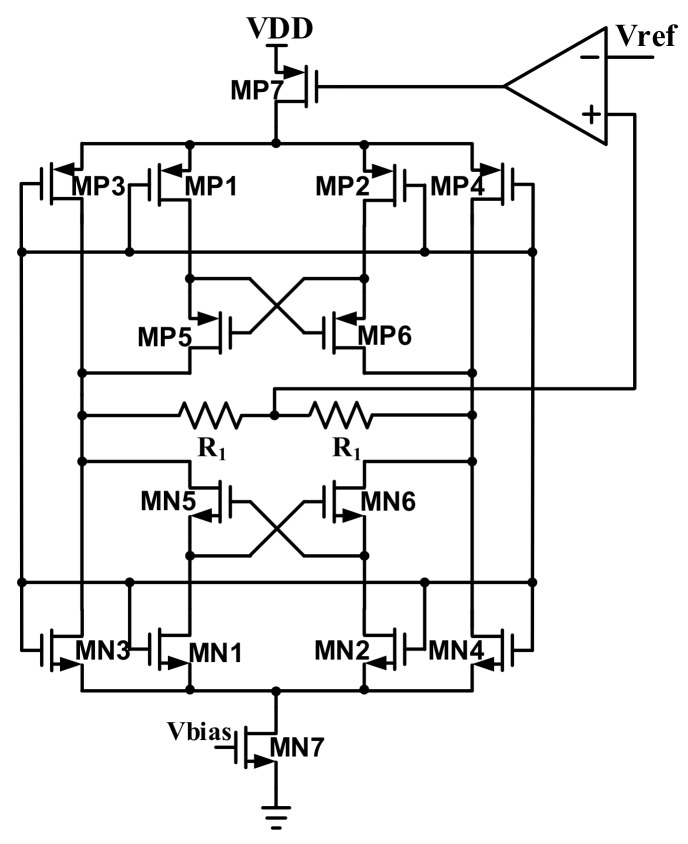
Architecture of the Gm cell.

Figure 16 presents the Gm cell that is used in the receiver. The cross-coupled Gm cell is adopted to cancel the third-order harmonic frequency. Therefore, it can acquire a high linearity and a current gain is obtained through gain boosting.

**Figure 17:**
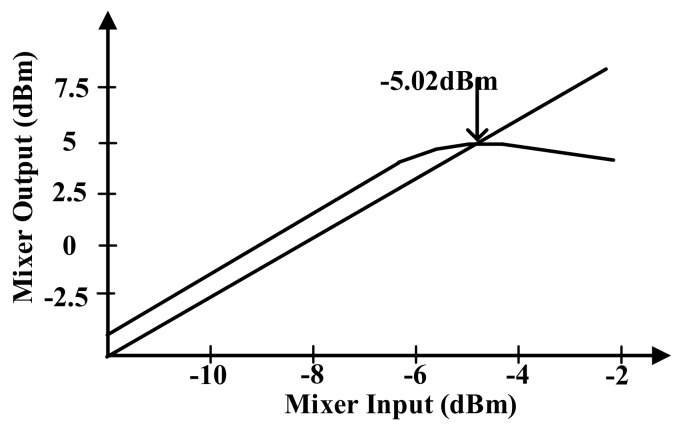
P1dB simulation of the receiver mixer.

Figure 17 shows the P1dB simulation result of the RX mixer. The designed RX mixer guarantees the linearity until −5.03 dBm.

### 3.6. Filter

**Figure 18:**
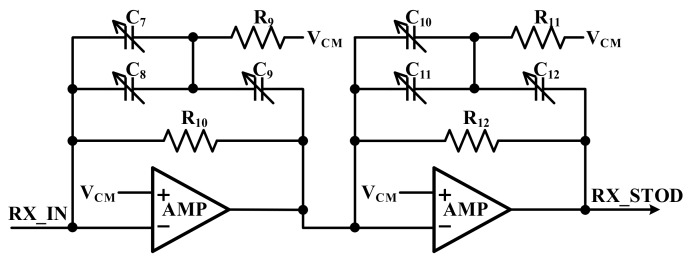
Block diagram of the high-pass filter.

Figure 18 shows the architecture of the HPF in the receiver. To reduce low-frequency charger noise, this block is the first of the AFE that is connected to the touch panel. The designed HPF is designed as fourth-order Butterworth, the touch signal is not affected, and the cut-off is approximately 300 kHz.

**Figure 19:**
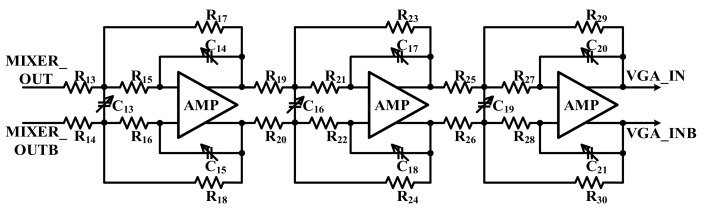
Block diagram of the low-pass filter.

Figure 19 shows the architecture of the LPF in the receiver. The role of the LPF in the RX elimination of the LO signal that remains in the touch signal. Since the down conversion frequency is under 300 kHz, the sixth-order Butterworth type is adopted and the cut off is designed at about 300 kHz.

**Figure 20:**
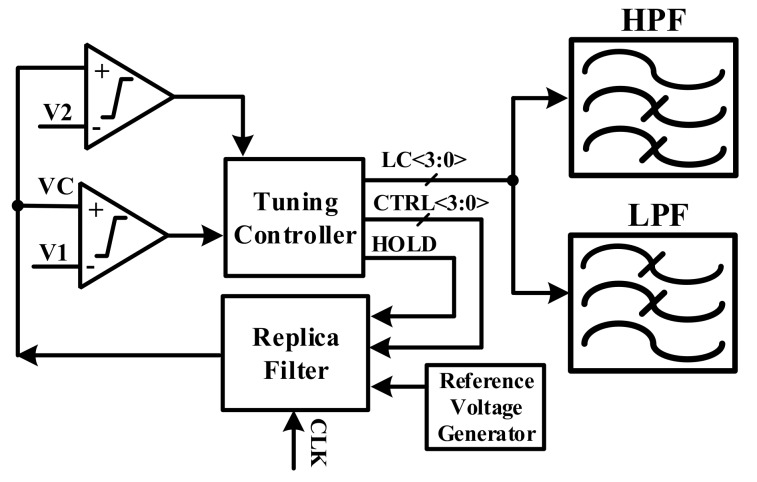
Block diagram of filter tuning.

Figure 20 shows the filter tuning block for the HPF and LPF. If the resistor and the capacitor are varied, the bandwidth of the HPF and LPF are changed. In order to minimize the capacitor and resister variations following the process, this block is designed to compensate the mismatch of the capacitor and the resistor. This block can also detect the process variation by using the replica filter. According to the value of the replica filter, this block changes the resisters and the capacitors of the HPF and the LPF. After the filter-tuning block calibrates the capacitor and the resister, the characteristics of each AFE channel must be constant.

## 4. Experimental Results

**Figure 21:**
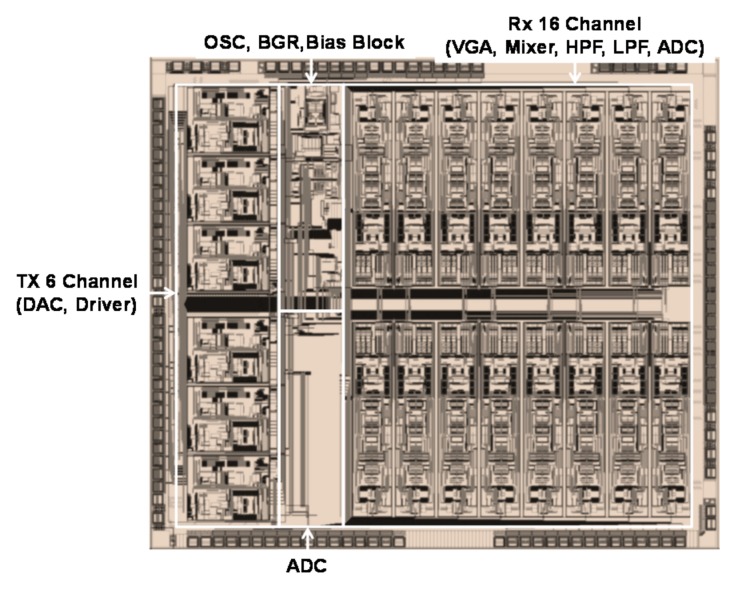
Chip microphotograph.

**Figure 22:**
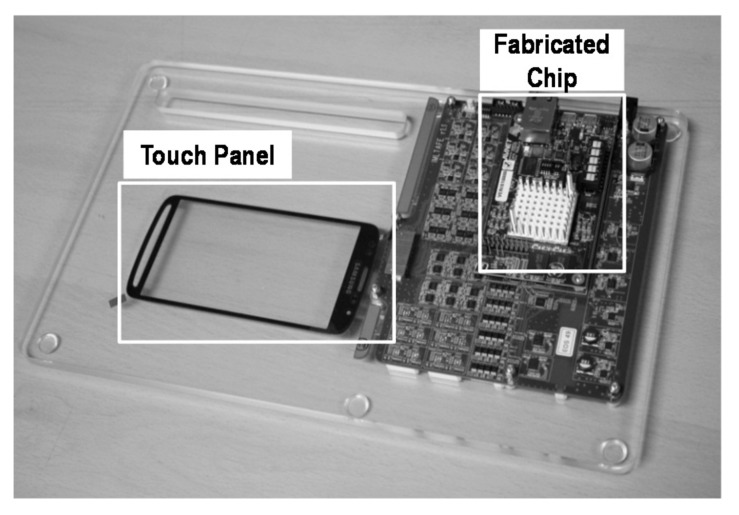
Measurement environment.

Figure 21 shows the chip microphotograph for the touch panel. To minimize the difference between the TX and the RX, symmetry is implemented. The chip area is 4.84 mm × 4.2 mm in the 90 nm CMOS process. Figure 22 shows the measurement test board for the AFE. It is composed of a mutual capacitance touch panel and fabricated chip for the verification of the performance of the AFE. The measured touch panel size is 5.1 inches.

**Figure 23:**
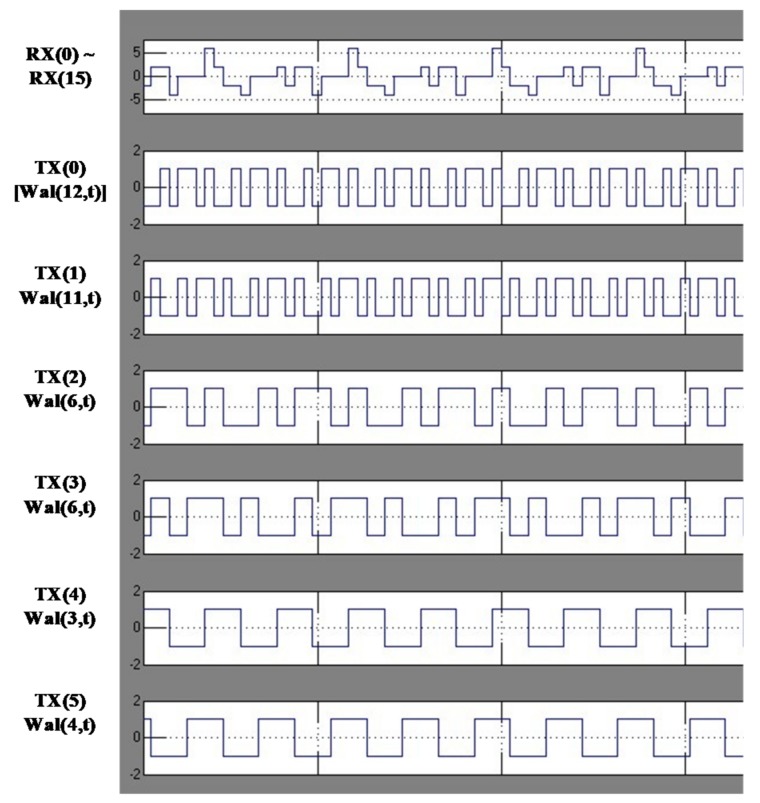
Transient simulation results of the 16th AFE channel.

Figure 23 shows the simulation results of the transmitter. Each transmitted signal from TX(0) to TX(5) in Figure 5. Each transmitted signal is added to the touch panel and the added signal is received in the RX. The frequency of characteristic of the signal from RX(0) to RX(15) is the same as those of each RX channel of the touch panel, with the magnitude representing the only difference. By using the orthogonality of each Walsh function, the touched location can be detected efficiently.

**Figure 24:**
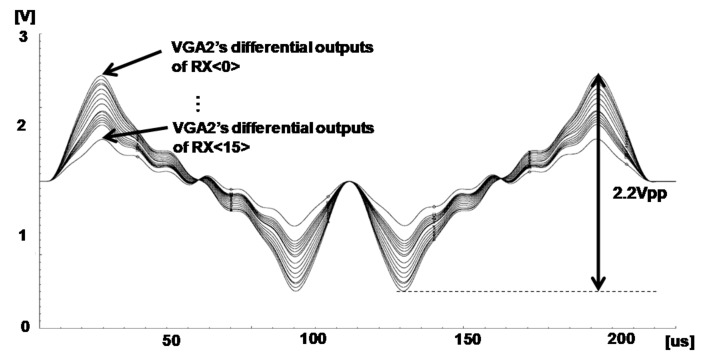
Transient simulation outputs of the VGA2 in the 16th RX AFE.

Figure 24 shows the transient differential outputs (VO-VOB) of VGA2 in the RX AFE 16th channel as shown in Figure 5. The 6th TX channel transmits the OFDM signal to the 16th RX AFE. Each carrier of the OFDM is combined in the touch panel and the 16th RX AFE receives the combined signal. The combined signals have a different amplitude as following the characteristic of the touch panel. Since each output of the VGA2 in the RX AFE has a different amplitude, this system can sense the position of the touch by using the order of the magnitude at each channel. If a touch is generated, the position is detected because the order of the magnitude from RX(0) to RX(15) is changed.

**Figure 25:**
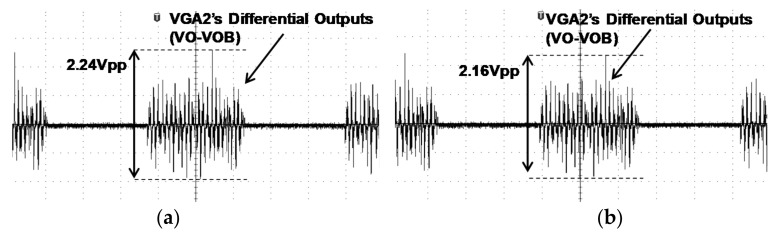
Measurement of (**a**) touch and (**b**) no touch OFDM signal.

Figure 25 shows the measurement results of VGA2’s differential outputs (VO-VOB) in Figure 5 when there are touch and no touch, respectively. When the touch is not applied on the touch panel, the VGA2’s differential outputs of RX(15) in the receiver is 2.24 Vpp, while the VGA2’s differential outputs of RX(15) is 2.16 Vpp when the touch is applied. The difference between the touch and no touch is approximately 0.1 V. Due to the generated touch, it can change the magnitude order, as shown in Figure 24.

**Figure 26:**
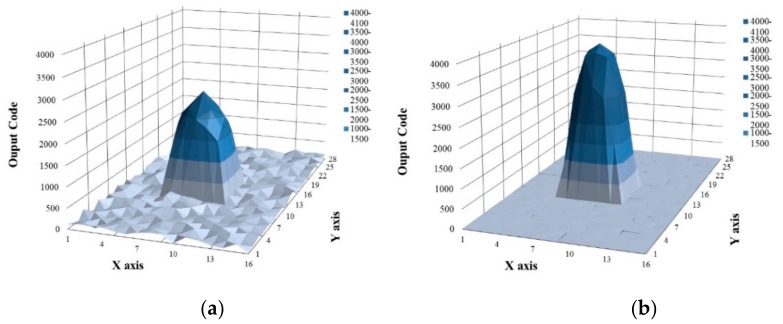
(**a**) Touch image before noise filtering, and (**b**) touch image after noise filtering.

Figure 26 shows the pen touch image before and after the noise filtering, respectively. The X and Y axes represent the channel of the touch panel. By using the output code, the touch spot can be detected. Figure 26a shows the touch image when the noise is not filtered. This case represents that the touch signal is passed without the HPF, LPF, and the mixer. Since the noise is not rejected and the touch signal is not upconverted, the noise is directly presented and the touch signal is reduced following the touch panel characteristic in Figure 3. Additionally, because the touch signal is not up converted, it is reduced by the characteristics of the panel, as shown in Figure 3. The SNR acquired is about 20 dB due to the noise and decreased touch signal. Figure 26b shows the touch image after filtering the noise. The noise can be rejected because the HPF, LPF, and the mixer are used. In addition, the characteristic of the panel is not affected because the signal is upconverted by the mixer.

**Figure 27:**
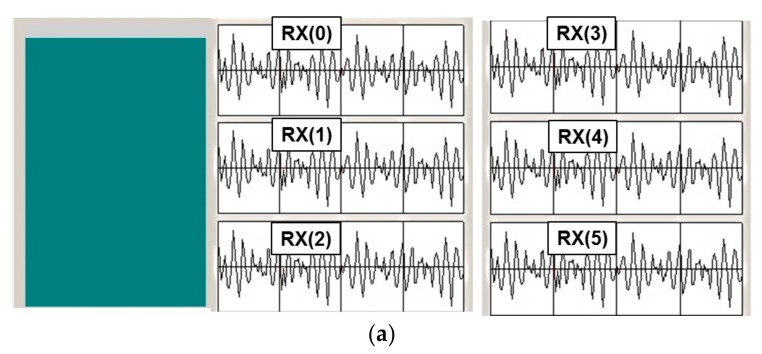

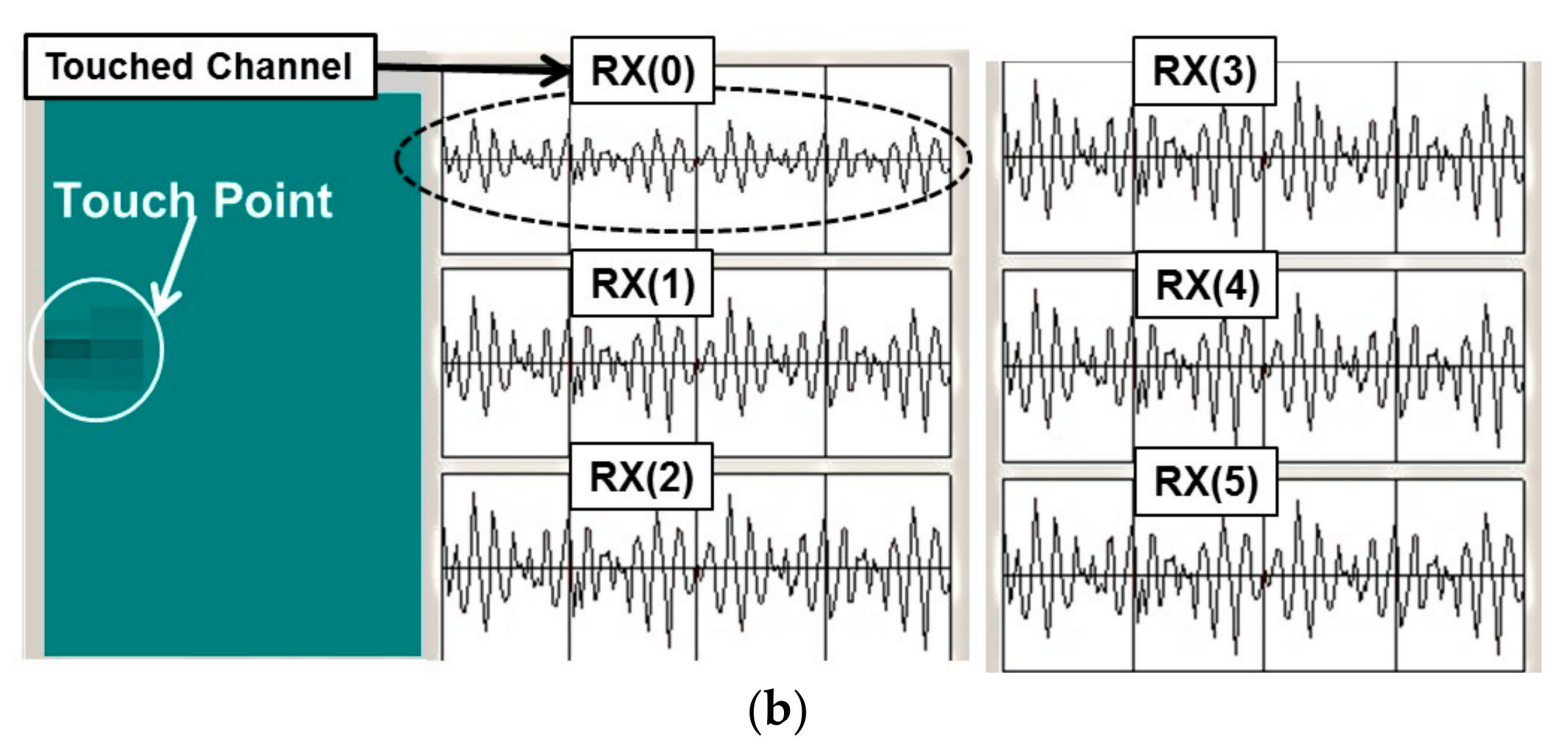
(**a**) No touch and (**b**) touch image in the software program.

Figure 27 shows the no touch and touch images in the software, respectively. By using the touch software, we can check the proposed design detects the position. Also, the signal of 6 receiver channel as shown in Figure 5 is presented in this program. We can perceive the touch point through the variation of the magnitude. If the touch is not generated, the software shows the same color, as shown in Figure 27a, whereas if the touch-point is generated, the color is changed, as shown in Figure 27b. The designed system can find the touch position exactly.

The SNR for the touch panel application is calculated from Equation (4) [19], as follows:

(4) $$\begin{array}{c} \mathrm{SNR}=20\times \log\frac{\left|M_{N}-M_{T}\right|}{\sigma }\\ M_{N}=mean\;value\;when\;not\;touched\\ M_{T}=mean\;value\;when\;touched\\ \sigma =standard\;deviation\;when\;touched \end{array}$$

The X axis and the Y axis represent the number of samples and the output code, respectively. To acquire the SNR, the mean values of the touch and no touch are calculated. In addition, the standard deviation of noise in touch is calculated. By subtracting the average of no touch from the average of touch, and then dividing this result by the standard deviation of the noise of touch, the SNR is acquired.

Table 1 shows the circuit performance summary of the proposed AFE for the touch panel. The application of this work is for a 28 × 16 channel capacitive touch panel. The power consumption is lower than those presented in [12,20,21]. The chip area is smaller than the reported results [12,21,22]. To obtain a high SNR and scan rate, the OFDM sensing scheme is proposed and the SNR of this chip attains up to 60 dB. Contrary to the conventional structure for the touch panel in [12,20,21,22], the mixer, VGA, and HPF are proposed in the AFE. The AFE of [12,20,21,22] is composed of integrators, but this paper proposes the mixer and VGA instead of integrators. Therefore, the touch signal processing time in the AFE is faster and higher than in [12,20,21,22]. Therefore, the proposed structure has a SNR of 60 dB and a frame rate of 200 Hz to scan the 28 × 16 channel capacitive touch panel.

## 5. Conclusions

In this paper, the AFE for a capacitive touch panel is presented. A high noise immunity, 28 × 16-channel finger touch sensing IC for an OFDM touch sensing scheme is presented for AFE. In order to increase the SNR, the OFDM sensing scheme is proposed. The TX transmits the orthogonal signal to each channel of the panel. The RX detects the magnitude of the orthogonal frequency to be transmitted from the TX. Due to the orthogonal characteristics, it can be robust to the narrowband interference and noise. Therefore, the SNR can be improved. To reduce the noise effect of low frequencies, a mixer, and a high pass filter are also proposed. After the noise is filtered, the touch SNR attained is 60 dB compared to 20 dB before the noise is filtered. The advantage of the proposed OFDM sensing scheme is its ability to detect channels of the panel simultaneously with the use of multi-carriers. To satisfy a linearity of the signal in the OFDM system, a high linearity mixer and a rail-to-rail amplifier in the TX driver are designed. The proposed design is implemented in the 90 nm CMOS process. The SNR is approximately 60 dB. The area is 13.6 mm^2^, and the power consumption is 62.4 mW.

## Figures and Tables

**Table 1:** Performance of the analog front end for the touch panel.

	[12]	[22]	[20]	[21]	This Work
Touch Panel Type	Mutual Capacitive	Mutual Capacitive	Mutual Capacitive	Mutual Capacitive	Mutual Capacitive
Touch Panel Channel	30 × 24	48 × 32	12 × 16	112 × 198	28 × 16
Technology	CMOS 180 nm	BCD 180 nm	CMOS 350 nm	CMOS 130 nm	CMOS 90 nm
SNR	39 dB	49 dB	27.5 dB	45.5 dB	60 dB
Frame Rate	240 Hz	120 Hz	175 Hz	977 Hz	200 Hz
Power Consumption	52.8 mW	30 mW	76 mW	797.4 mW	62.4 mW
Area	14.8 mm^2^	14.7 mm^2^	5.02 mm^2^	74.17 mm^2^	13.6 mm^2^
